# Dissecting biomarker networks linking COVID**-**19 inflammatory drivers, disease severity and thyroid adaptive responses

**DOI:** 10.1038/s41598-025-25434-1

**Published:** 2025-11-24

**Authors:** Assem Aimaganova, Natalia Khovanova, Emma Braybrook, Evangelos Vryonis, Neil R. Anderson, Lawrence Young, Dimitris K. Grammatopoulos

**Affiliations:** 1 School of Engineering, University of Warwick, Coventry, CV4 7AL UK; 2 Institute of Precision Diagnostics and Translational Medicine, University Hospitals Coventry and Warwickshire NHS Trust, Coventry, UK; 3 Division of Translational and Experimental Medicine, Warwick Medical School, University of Warwick, Coventry, CV4 7AL UK

**Keywords:** Interleukin**-**6, Thyroid, TSH, Free T3, Inflammation, Critical care, Biomarkers, Diseases, Endocrinology, Immunology, Medical research

## Abstract

Altered thyroid function (TF), especially non**-**thyroidal illness syndrome (NTIS), is common in hospitalised COVID**-**19 patients, but adaptive responses to disease as captured by blood biomarkers in the context of thyroid signal integration require further elucidation. This retrospective study analysed 29 routine biomarkers, including immune, inflammatory, haematological, organ damage, and TF markers, from patients admitted to the University Hospital Coventry and Warwickshire, decoding associations of the biomarkers with TF key determinants and identifying patterns of severity**-**specific thyroid dysfunction. Patients were categorised by disease severity based on admission to either critical care units (CCU) or general wards. Among 237 records, 140 were CCU admissions (mean age 57, 69% male) and 97 were general ward patients (mean age 63, 59% male). Both groups exhibited negative correlations between haematology and TF biomarkers with inflammation and organ dysfunction markers. The pattern of clustering and the deduced network built around the correlation of TF components also exhibited disease severity**-**specific characteristics. At least 11 biomarkers, including free**-**triiodothyronine (fT3) and free**-**thyroxine (fT4), showed significant group**-**specific differences. Notably, CCU patients demonstrated an interleukin**-**6**-**dependent enhancement of correlations between TSH and fT3 or the fT3/fT4 ratio. Our findings suggest an immune-driven adaptation of the pituitary-thyroid axis, often presenting as NTIS in COVID-19. Deciphering information from routine biomarkers could enhance TF assessment, particularly in critically ill patients.

**Supplementary Information:**

The online version contains supplementary material available at 10.1038/s41598-025-25434-1.

## Introduction

Patients with SARS**-**CoV**-**2 infection can experience a range of clinical manifestations, from no symptoms to critical illness^1^. COVID**-**19 is a multisystem disease caused by a diffuse systemic process involving a complex interplay of immunological, inflammatory, and coagulative cascades. Severe or critical cases of COVID**-**19 might present with multi**-**organ involvement, including cardiovascular, gastrointestinal, nervous, and endocrine systems^2^. The ability of the virus to affect endocrine organs and physiological functions is facilitated by the widespread expression of angiotensin**-**converting enzyme 2 (ACE2) and transmembrane serine protease 2 (TMPRSS2) proteins on host cells that act as receptors for the virus^3,4^. It is generally thought that endocrine gland diseases linked to SARS**-**CoV**-**2 infection have multiple causes. These include a combination of direct infection of the endocrine gland by the virus, activation of the hypothalamic**-**pituitary axis by inflammatory mediators driving the ‘cytokine storm’, and end**-**organ damage induced by the immune response^2^.

Thyroid dysfunction is a common endocrinopathy in hospitalised COVID**-**19 patients^5^. The most common pattern of deranged thyroid function (TF) tests reported is reminiscent of non**-**thyroidal illness syndrome (NTIS) or sick**-**euthyroid syndrome^6^. This term describes a combination of TF abnormalities in the absence of intrinsic thyroid disease and is considered part of the type 1 allostatic load responses^7^. It usually presents with lower or normal thyroid**-**stimulating hormone (TSH) levels and, in many cases, decreased triiodothyronine (T3) concentrations^8^. Several studies suggested that the degree of decrease in TSH and/or T3 was positively correlated with the severity of the COVID**-**19 disease, and in particular, free T3 (fT3) concentrations were significantly lower in the deceased ones^9–12^.

NTIS can occur in several acute or chronic systemic diseases among hospitalised patients, infectious diseases, sepsis, and hospitalisation in the critical care units (*CCU)*^13,14^. It is thought that NTIS identifies the activation of homeostatic adaptation mechanisms, such as those seen in starvation, to enable recovery^14^ from critical illness by limiting the availability of the active T3 and, thus, decreasing energy expenditure and limiting catabolism. However, it is possible NTIS represents a maladaptive mechanism. Recent evidence suggests that the pathogenesis of NTIS is complex and includes both peripheral and central components^15,16^. In conditions where NTIS is associated with excessive secretion of inflammatory mediators, such as COVID**-**19, cytokines appear crucial in driving dysregulation of the hypothalamic**-**pituitary**-**thyroid axis^17^ through (a) altering hypothalamic ‘set**-**points’ that initiate thyrotropin**-**releasing hormone release in response to low T3 levels by up**-**regulating the hypothalamic deiodinase enzymes D1 and D2, which convert thyroxine (T4) into T3; (b) suppressing pituitary TSH secretion and reducing iodine uptake and thyroid hormone excretion; (c) down**-**regulation of the deiodinase enzymes (especially liver D1 activity) that convert T4 into T3 and the up**-**regulation of the enzymes such as D3 that convert thyroid hormones into the inactive metabolite reverse T3 (rT3)^16^.

Much of COVID**-**19 research has focused on the impact of the pleiotropic cytokine interleukin**-**6 (IL**-**6) on thyroid hormone output and clinical outcomes^18–20^. However, an integrative approach exploring the distinct interconnectivity of routine TF tests, such as TSH, fT3, and free T4 (fT4) and collective responses to hyper**-**inflammatory states can provide a more comprehensive understanding of the thyroid adaptive dynamics observed in inflammatory diseases like COVID**-**19.

Since IL**-**6 levels have consistently been identified as a strong prognostic indicator of severe disease in hospitalised COVID**-**19 patients^21^, this study explored correlations between common routine biomarkers of patients from two distinct settings characteristic of disease severity [general *wards* and *CCU*], focusing on TF tests. Instead of clinical outcomes, we focused on biomarker network dynamics to decipher patterns associated with disease severity and how this affects thyroid biomarker interactions and adaptive responses.

## Results

### Patient data

For this observational cohort study, retrospective COVID**-**19 biomarker data from 575 laboratory requests of 472 COVID**-**19 patients were extracted from the University Hospital Coventry and Warwickshire (UHCW) NHS Trust Pathology laboratory information system. The mean age of the cohort was 63.7 ± 16.8 years; 42.4% female. 42.1% of biomarker datasets were from patients in the *CCU*. Data included demographic information (age, sex), clinical request details such as patient location, and COVID**-**19 biomarker panel blood results taken during hospitalisation of patients with a positive polymerase chain reaction (PCR) result. The biochemistry COVID**-**19 biomarker panel was used, according to hospital protocols, in patients with active infections. This panel included biomarkers recommended by RCPath in April 2020^22^ and employed to monitor disease severity and progression. The characteristics of the reviewed biomarkers are given in Supplemental Table S1. Biomarker data descriptions and analysis methods are presented in the Supplemental Information file.

The UHCW NHS Trust COVID**-**19 research committee exempted this study from ethics oversight as the main purpose was to gather data regarding biomarker associations, laboratory parameters, and disease outcomes.

### Biomarker profiling in the ward and CCU groups

The *ward* and *CCU* location split was 59% and 41%, respectively. The *CCU* group included.

mostly younger male patients (57 y.o. vs 63 y.o.; males 69% vs 59%) who exhibited significantly higher rates of anaemia (62% vs 46%) (Supplemental Table S2). Table 1 lists the biomarkers that showed significant differences in the *ward* and *CCU* groups. Patients in the *CCU* group had significantly lower and below the reference range haematological indices: red blood cells (RBC), haemoglobin (HB) and haematocrit (HCT), suggesting anaemia, as well as albumin (ALB) and fT3 (Table 1). Biomarkers of inflammation, tissue damage, and cell death, such as IL**-**6, ferritin (FER), procalcitonin (PCT), troponin (TNT), urea, and lactate dehydrogenase (LDH), were significantly different compared to the *ward* non**-**severe group and well above the reference range. This biomarker picture of raised LDH, ferritin, urea, and TNT, but lower HB and albumin, has been extensively described in COVID**-**19 patients requiring admission to the *CCU*^23,24^.

**Table 1:** Biomarker values compared to local laboratory reference ranges. The green area indicates the laboratory population reference range; the grey line signifies the range across the cohort; the blue and red triangles indicate group median values. ^*1*^CWPS , *Coventry and Warwickshire Pathology Services *;^*2*^*Post-menopausal female; *^*3*^*Pre-menopausal female.*

**Biomarker name**		*p*-value	**CWPS**^**1**^** reference range**	***Ward* median**	***CCU* median**	
RBC (× 10^12^/L)	Male	0.01	4.50–5.30	4.39	3.83	
	Female	0.3	4.10–5.10	4.27	4.07	
HB (g/L)	Male	0.02	130–170	130	110	
	Female	0.1	120–150	121	118.5	
HCT (L/L)	Male	0.01	0.40–0.50	0.40	0.35	
	Female	0.04	0.36–0.46	0.38	0.36	
ALB (g/L)		< 0.001	35–50	35	31	
FT4 (pmol/L)		< 0.001	12–22	17.6	15.5	
FT3 (pmol/L)		< 0.001	3.1–6.8	3.7	2.8	
IL-6 (ng/L)		< 0.001	< 8	28	128	
FER (mg/L)	Male Female^2^	0.02	15–350	699	955	
	Female^3^	0.7	10–150	731	670	
PCT (ug/L)		0.002	< 0.06	0.13	0.22	
TNT (ng/L)		0.008	< 14	16	25	
UREA (mmol/L)		< 0.001	2.5–7.8	6.9	10	
LDH (U/L)		< 0.001	0–250	381	495	

### Biomarker correlations analysis

The initial biomarker cluster map for the whole cohort, using Spearman correlation coefficients, included 31 biomarkers from Supplemental Table S1 and all available samples (575), and identified three biomarker clusters that contained haematological, inflammatory, and immune cell biomarkers. The first cluster included haematology biomarkers (RBC, HB, HCT, transferrin, albumin, iron) and thyroid hormones (fT3, fT4). The second cluster included inflammation biomarkers (IL6, ferritin, neutrophil**-**to**-**lymphocyte ratio (NLR), PCT, C**-**reactive protein (CRP)), kidney function biomarkers (urea, creatinine (CRE)), as well as TNT, LDH and RBC distribution width (RDW). The third cluster included mean corpuscular volume (MCV), mean corpuscular haemoglobin (MCH), sodium, potassium, white cell count (WCC), platelets, monocytes, eosinophils, basophils, and TSH. The first and second clusters demonstrated well**-**defined patterns, where biomarkers *within* each cluster correlated positively. In contrast, biomarkers *from* the two clusters correlated negatively with each other. The biomarkers within the third cluster did not have any pattern, correlating moderately only in pairs as MCV with MCH (*ρ* = 0.76), monocytes with WCC (*ρ* = 0.49), and eosinophils with basophils (*ρ* = 0.4), so 9 biomarkers from the third cluster, other than TSH, were excluded from further consideration.

**Fig. 1:**
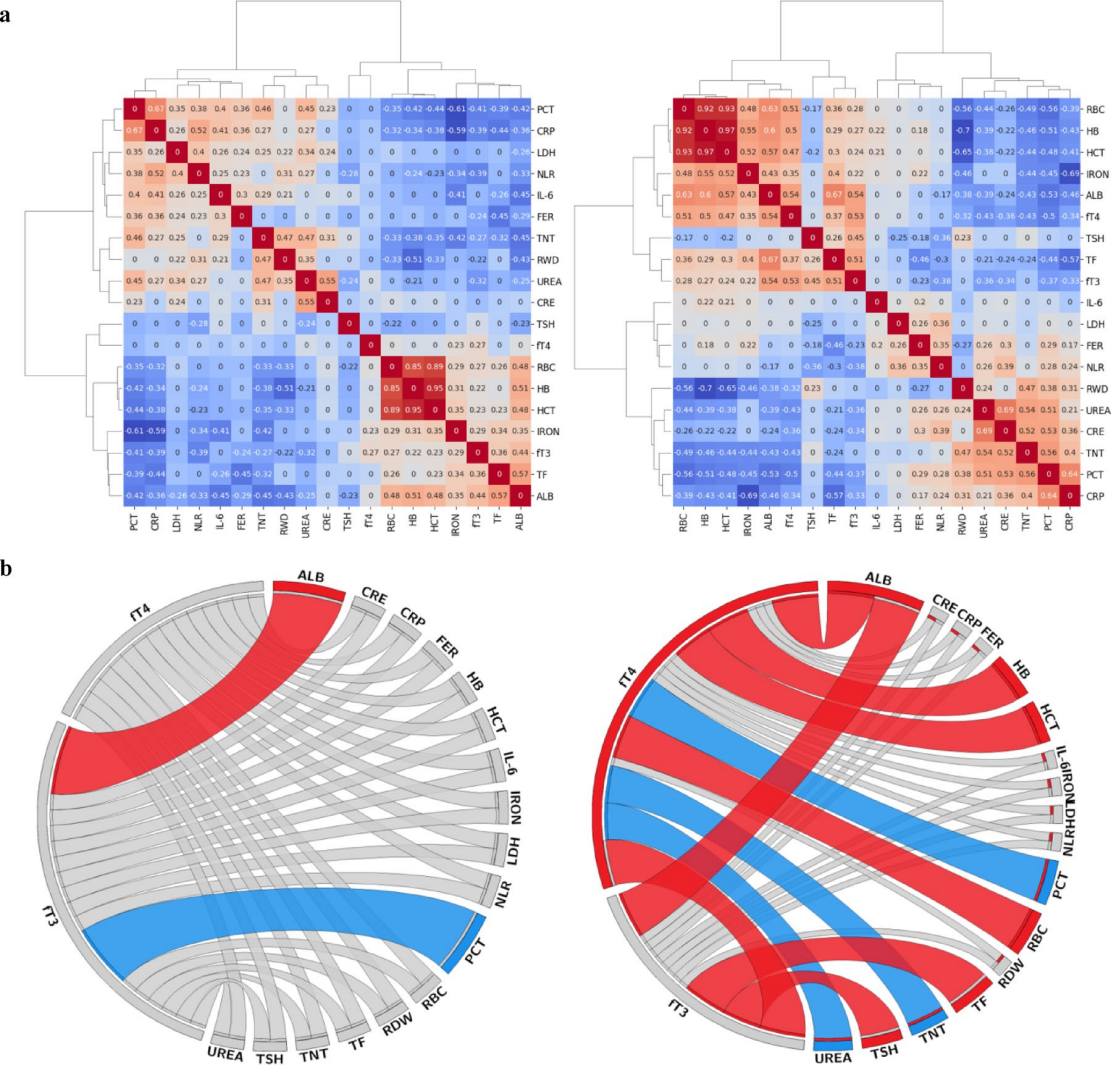
Correlation patterns and thyroid-related biomarker networks in *ward* and *CCU* patients. (**a**) Correlation heat maps with dendrograms show biomarker clusters in *ward* (left) and *CCU* (right) patients (237 samples, 19 biomarkers). Red: positive correlations; blue: negative. (**b**) Circos diagrams show correlations of biomarkers with TF markers. Positive correlations (ρ ≥ 0.4): red; negative (ρ ≤ -0.4): blue.

Next, we repeated group**-**specific (non**-**severe *ward* vs *CCU*) biomarker correlations analysis to generate cluster maps with hierarchical clustering dendrograms for 19 biomarkers (Fig. 1a). The *CCU* group contained two clusters of positive and negative correlations, shared by the haematology and inflammation/organ dysfunction biomarker groups. In contrast, in the *ward* cohort, the correlation strength of these clusters appeared weaker and less coherent compared to the *CCU*, based on the number of moderate/strong correlations present.

Overall comparison of biomarker correlations with moderate/strong correlation coefficient (*ρ* ≥ 0.4) identified significant differences between the two groups (Supplemental Fig. S1), revealing a more synchronised response in the face of severe disease. Potential biomarker networks that specifically correlate with components of the pituitary**-**thyroid (P–T) axis, namely TSH, fT4, and fT3 were visualised by circos diagrams (Fig. 1b), with only moderate/strong correlations (*ρ* ≥ 0.4) included.

### Thyroid function biomarker correlations in the ward and CCU groups

One of the notable differences in biomarker levels between the two patient groups was in fT3 levels (Table 1), so we focused on P–T hormone correlations. Although, in all**-**patient data analysis, TSH vs fT3/fT4—a marker of conversion of T4 to T3—exhibited weak correlations (*ρ* < 0.4), a different picture emerged when data were split according to disease severity. Specifically, plotting TSH vs fT3/fT4 showed that in the majority of *ward* patients, TSH was within the reference range, and only a few patients had fT3 values below the reference range. In contrast, in the *CCU* group, more than half of the points had a normal TSH with fT3 below the reference range, pointing towards a significantly higher proportion of NTIS cases in the severe disease group (Supplemental Fig. S2a). In a small subset of cases, fT4 was also below the reference range (Supplemental Fig. S2b).

**Fig. 2:**
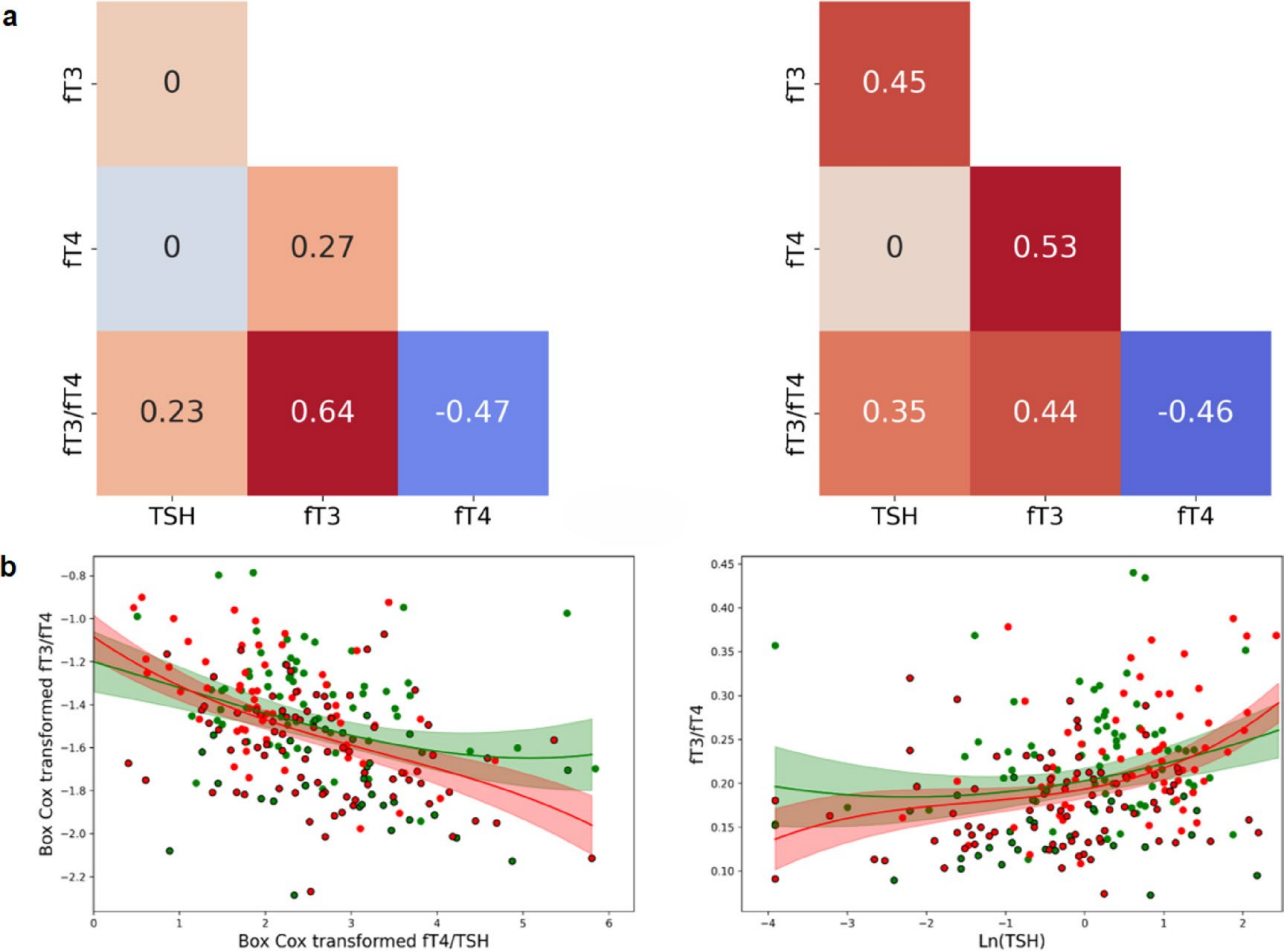
Thyroid function marker correlations and nonlinear relationships in *ward* and *CCU* patients. (**a**) Correlation coefficients of TF markers in the *ward* (left) and the *CCU* (right). Zero indicates non-significant correlations. (**b**) Scatter plots of fT3/fT4 vs fT4/TSH and fT3/fT4 vs ln(TSH). Green: *ward*; red: *CCU*; circled: low fT3 (below reference range). Curves fitted by a third-degree polynomial. Shaded areas: 95% confidence limits.

To characterise interactions of all components of the P–T axis, we profiled correlations across these biomarkers. In *ward* patients, there was no moderate or strong correlation between TF biomarkers (Fig. 1b). In contrast, in the *CCU* group, moderate to strong correlations were observed between TSH and fT3, as well as between fT4 and fT3, identifying a coordinated response of the P–T axis in *CCU* patients. Further analysis (Fig. 2a) identified no correlation between TSH and fT3 in the *ward* group, but a moderate correlation between these two biomarkers in the *CCU* group with *ρ* = 0.45. A similar analysis in the fT3**-**fT4 relationship between the two groups also identified significant differences in correlations: *ρ* = 0.27 in the *ward* group and *ρ* = 0.53 in the *CCU* group.

To obtain information about the ‘relational stability’ of the P–T axis^25^ and describe the adaptive inter**-**relationships between thyroid hormone parameters, the fT3/fT4 vs fT4/TSH ratios were plotted (Fig. 2b) as indicators of pituitary influence on thyroid hormonal release and conversion of fT4 to the active hormone fT3. Interestingly, despite differences in individual correlations between thyroid hormones, both patient groups exhibited similar profiles of continuous inverse responses.

### Thyroid biomarker correlations: influence by inflammatory mediators

**Fig. 3:**
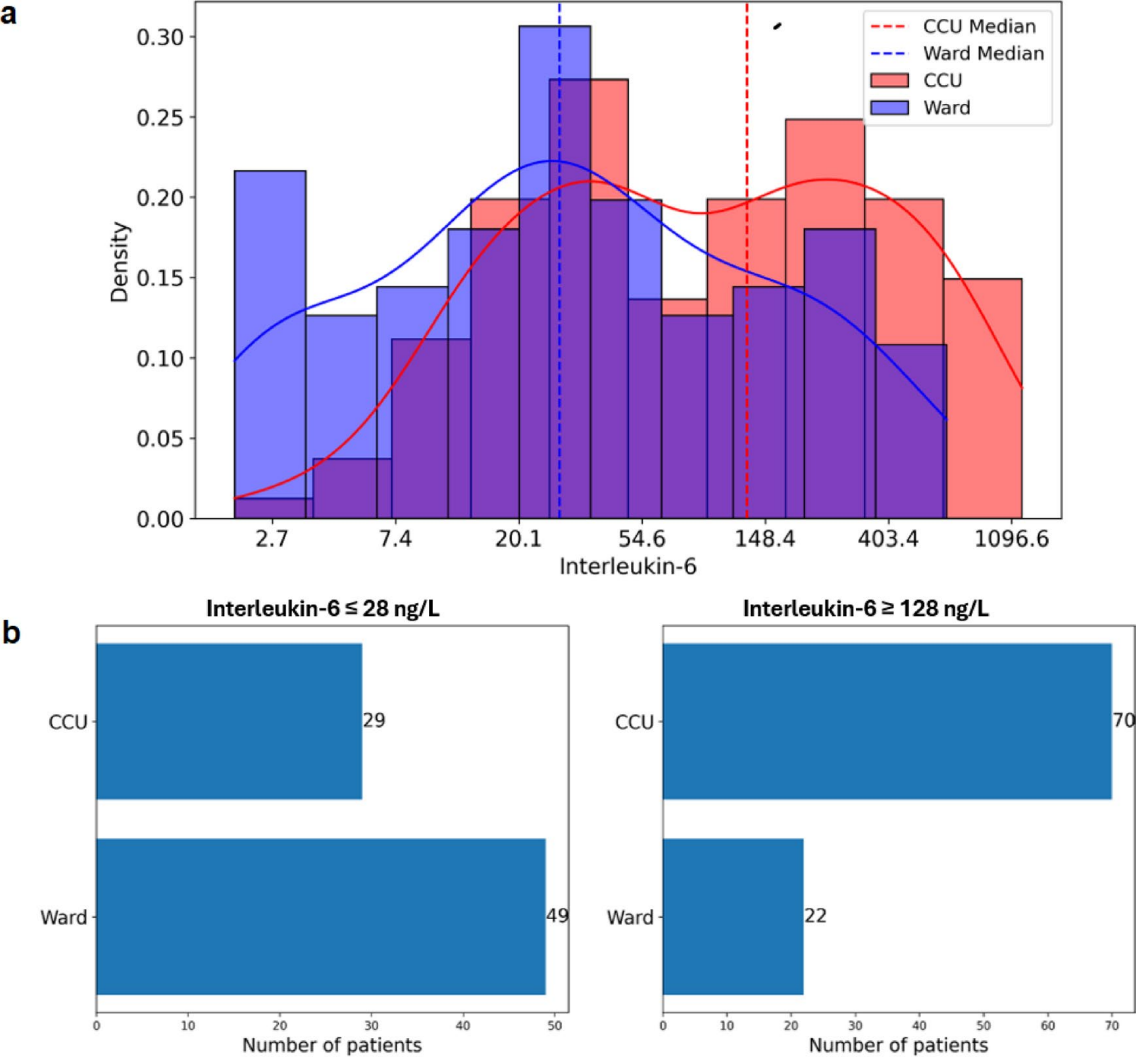
Distributions of IL-6 levels in *ward* and *CCU* patients. (**a**) Log-transformed IL-6 distributions with density curves (solid) and medians (dashed) for *ward* and *CCU*. (**b**) Distribution of samples split by IL-6 ≤ 28 ng/L (left) and IL-6 ≥ 128 ng/L (right).

We explored mechanistic insights into the biomarkers potentially associated with different patterns of thyroid biomarker correlations between the two disease severity groups. The initial focus was on IL**-**6, which has a well**-**described effect on hypothalamic**-**pituitary**-**thyroid function^26^ and also exhibited significant differences in the median levels between subgroups in our study (Table 1): 28 ng/l in the *ward* group and 128 ng/l in the *CCU* group. Distributions of IL**-**6 values with density curves and median lines demonstrated partial overlap between the *ward* and *CCU* groups and a wider spread of values in the *CCU* group towards the right**-**high values end of the distribution (Fig. 3a).

**Fig. 4:**
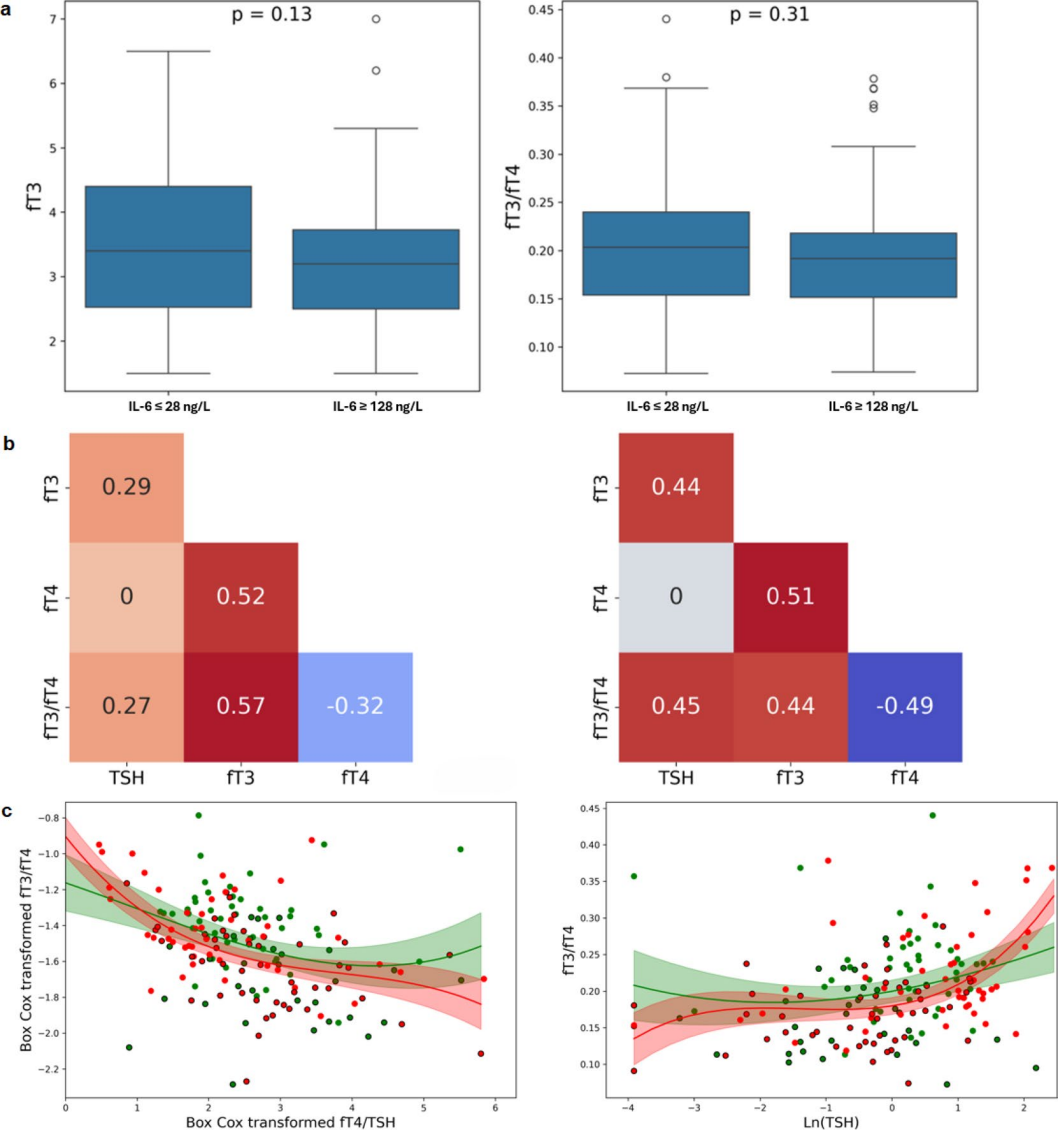
Thyroid function marker profiles and correlations at low and high IL-6 levels. (**a**) Distributions of fT3 and fT3/fT4 by IL-6 ≤ 28 ng/L and IL-6 ≥ 128 ng/L. (**b**) TF biomarker correlation maps for IL-6 ≤ 28 ng/L (left) and IL-6 ≥ 128 ng/L (right). (**c**) Scatter plots of fT3/fT4 vs fT4/TSH and fT3/fT4 vs ln(TSH). Green: IL-6 ≤ 28 ng/L; red: IL-6 ≥ 128 ng/L.

We then investigated whether differences in IL**-**6 levels, independent of the patient location, can explain different patterns of correlations across the TF test. This part of the study focused on biomarker datasets with IL**-**6 values of ≤ 28 ng/l or ≥ 128 ng/l, the cut**-**off values selected based on the medians of the two disease severity groups (Table 1). There were 78 samples with IL**-**6 ≤ 28 ng/l, and 37% of them were from the *CCU* (Fig. 3b); and 92 samples had IL**-**6 ≥ 128 ng/l, where 76% were from the *CCU*. These two groups also had similar percentages of anaemic patients (57%). Moreover, in these two groups, the distributions of fT3 and fT3/fT4 were comparable, and no significant differences were identified (*p* = 0.1 and *p* = 0.3, respectively) (Fig. 4a).

The TF correlation grids of the two groups (IL**-**6 values either ≤ 28 ng/l or ≥ 128 ng/l) (Fig. 4b) were comparable with those of the *ward* and *CCU* groups (Fig. 2a), with one notable exception: in the IL**-**6 ≤ 28 ng/l group, fT4**-**fT3 exhibited moderate correlation unlike the *ward* group, where the correlation was weak *(ρ* = 0.52 vs* ρ* = 0.27). Plotting the fT3/fT4 vs fT4/TSH ratios in the two groups (high or low IL**-**6) (Fig. 4c) demonstrated similar responses as shown in Fig. 2b, identifying the ability of the P–T axis to adapt to raised IL**-**6 levels and altered fT3/fT4 ratios by activating pituitary effects on thyroid hormonal release.

**Fig. 5:**
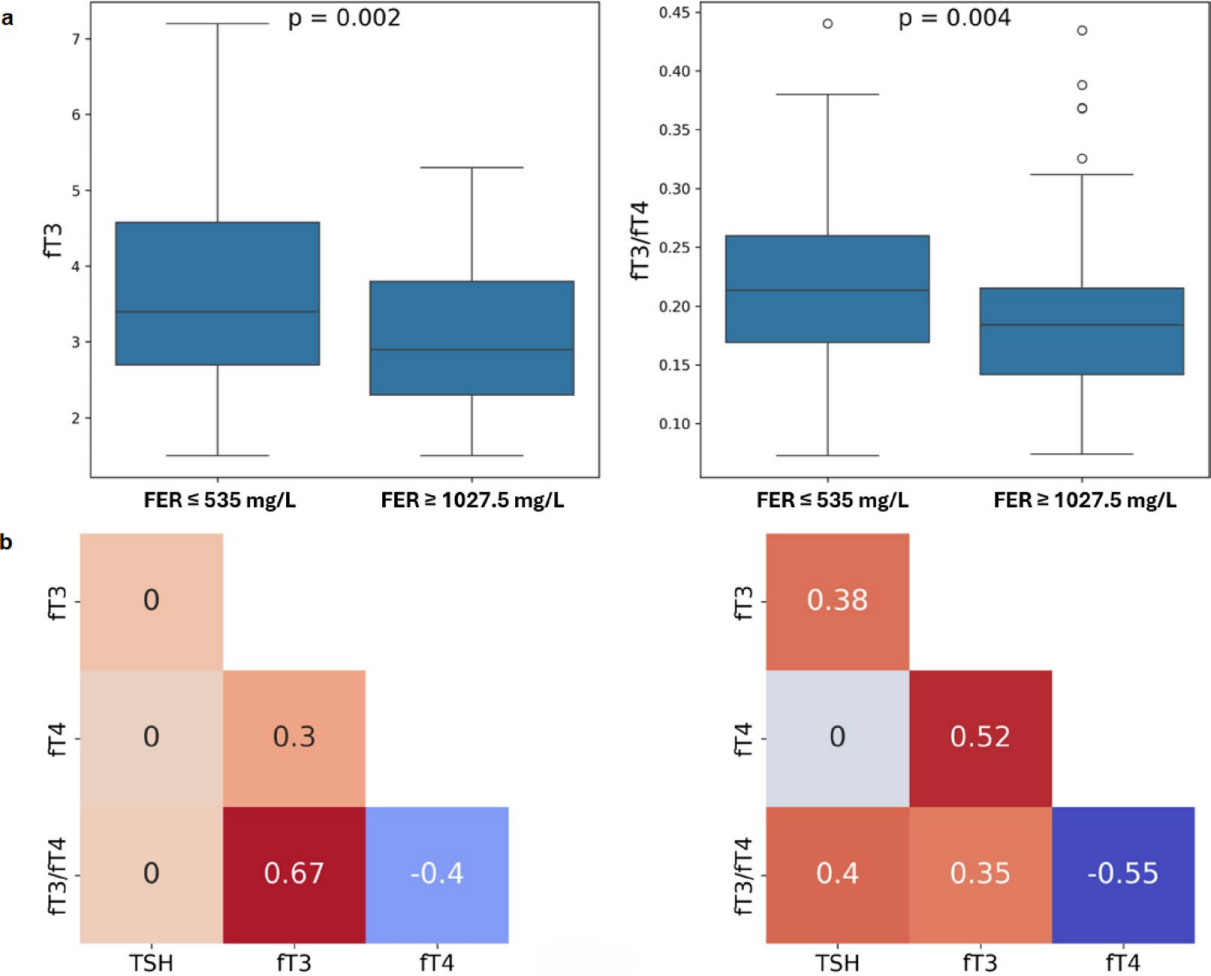
Thyroid function marker distributions and correlations at low and high ferritin levels. (**a**) Distributions of fT3 and fT3/fT4 in groups with ferritin ≤ 535 mg/L and ferritin ≥ 1027.5 mg/L. (**b**) TF biomarker correlation coefficients for ferritin ≤ 535 mg/L (left) and. ferritin ≥ 1027.5 mg/L (right).

One notable characteristic of the two ‘extreme’ groups with IL**-**6 values (either ≤ 28 ng/l or ≥ 128 ng/l) was the concomitant differences in some inflammatory biomarkers, such as ferritin, but not others like CRP. Ferritin, an acute phase reactant, was significantly lower in the IL**-**6 ≤ 28 ng/l compared to the IL**-**6 ≥ 128 ng/l group (median 535 ug/l vs 1027 ug/l). Using these ferritin values as cut**-**offs, we compared the distribution of fT3 and fT3/fT4, and identified significant differences (*p* = 0.002 and *p* = 0.004, respectively) (Fig. 5a). Moreover, the TF biomarkers correlation grid of the two ferritin groups (535 ug/l vs 1027 ug/l) identified correlations like those seen in the *ward*/*CCU* and IL**-**6 levels group analysis: moderate/strong correlations in TSH**-**fT3, TSH**-**fT3/fT4, and fT3**-**fT4 were observed only in the high ferritin (≥ 1027 ug/l) group (Fig. 5b).

## Discussion

Studies of patient abnormalities during COVID**-**19 identified a 26% prevalence of NTIS^6^ and the “cytokine storm” appears to be an important mediator^27^. Early studies of COVID**-**19**-**related thyroid abnormalities suggested that the development of NTIS may influence the biochemical presentation of thyrotoxicosis^28^. In addition, atypical painless thyroiditis was identified in some patients affected with severe COVID**-**19, characterised by mild thyrotoxicosis coexisting with NTIS^29^. In this study, we analysed real**-**world biomarker data, aiming to understand TF dynamics and responses to disease signals arising from distinct pathogenic patterns, such as hyperinflammation due to cytokine storm, a key feature of severe disease^2,30^. Instead of correlating absolute levels of biomarkers with disease severity and outcomes, an approach widely used in similar studies, we systematically explored patterns of correlations across different biomarkers, focusing on biomarker networks, an approach that can provide unique information about an organism’s systemic responses according to disease severity.

The two groups of patients exhibited distinct patterns of biomarkers characteristic of disease severity as previously described^31^ *CCU* patients exhibited raised LDH, ferritin, urea, and TNT, but lower HB and albumin; the latter has previously been shown to be negatively associated with acute mortality from COVID**-**19^32^. Moreover, one of the most striking characteristics of the two disease severity groups was the differences in IL**-**6 levels, which is considered the master regulator of COVID**-**19 severity biomarkers^33^. The presence of a ‘dense’ biomarker network of enhanced correlations in *CCU* patients compared to the *ward* group suggests activation of coordinated responses associated with enhanced disease severity that likely reflect the intensity or the duration of the underlying disease. Two biomarker groups (haematology and inflammation/organ dysfunction biomarkers) with positive intra**-**cluster and negative inter**-**cluster correlations appear to reflect these responses. The intensity and density of these correlations were diluted and scattered in *ward* patients, although the type of participating biomarkers was not altered.

Biomarkers of the P–T axis represented a key component of this dynamic biomarker network that covers a range of inflammatory and organ dysfunction biomarkers. A key finding in *ward* patients was the negative association between fT3, but not fT4, and NLR, which, in turn, showed positive correlations with inflammatory markers, CRP, and PCT. Similar associations between lymphopenia and fT3 have been reported^34^, and patients who had both lymphopenia and NTIS were more likely to have severe COVID**-**19 outcomes. Some common moderate/strong biomarker correlations were also present; one example is fT3 and albumin, which have previously been used in mortality prediction studies^35^. Serum albumin, which was previously shown to be negatively associated with acute mortality from COVID**-**19^36^ demonstrated a significant correlation with fT4 in the *CCU* group, but not the *ward* group, as previously described^37^.

In patients with severe disease, this P–T biomarker module is strengthened and enriched with at least 50% additional correlations. This suggests activation of synchronised (or perhaps distorted) adaptive responses in *CCU* patients, reflected by enhanced biomarker correlations with TF hormones interacting with specific haematological and inflammation/organ dysfunction biomarkers. The NTIS picture of low fT3 and normal TSH and fT4 was dominant in the *CCU* subgroup. As previous studies have linked thyroid dysfunction with disease severity and poor outcomes^4,6,20^, our studies expand this, providing a biomarker network template of thyroid dysfunction in severe cases involving haematological markers, markers of organ dysfunction, inflammatory markers, and targets of tissue damage.

In euthyroid patients, the concept of ‘relational stability’ is primarily driven by prioritising T3 stability and parallel control of the normal TSH**-**T4**-**T3 homeostatic set points^25^. It is thought that TSH exerts a feedforward control over the deiodinase activity that increases peripheral conversion of T4 to T3 and thus stabilises circulating fT3 levels. Perturbations of this homeostatic equilibrium in conditions such as NTIS lead to set**-**point adjustment and alterations in peripheral transfer parameters of the P–T control loop. The NTIS is observed in critically ill patients in *CCU* and is considered an adaptive metabolic response and a consequence of the acute phase stress response to systemic illness and macronutrient restriction, which might be beneficial for survival^14^, as it would restrict catabolism via decreased thyroid hormone action in important T3 target organs, such as the liver and muscle. In cases of COVID**-**19 associated with increased release of inflammatory mediators such as cytokines, dysregulation of the hypothalamic**-**pituitary**-**thyroid axis is thought to be mediated via deranged feedback regulation of the axis that includes altered hypothalamic ‘set**-**points’ that initiate thyrotropin**-**releasing hormone release in response to low T3 levels; and changes in the central regulation of the thyroid axis, including decreased TSH pulsatility and changes in the peripheral components of the thyroid axis^17,38^. Iron deficiency anaemia might also contribute, as deiodinases, which are iron**-**dependent, become less active, reducing the production of thyroid hormone^39^.

By examining the P–T axis homeostatic responses through systematic mapping of hormonal biomarker correlations, we were able to identify discrete patterns of correlations in each group of patients; in the *ward* patients, hormonal equilibria between TSH, fT4, and fT3 exhibited weak or no correlations, as previously described^40,41^. In contrast, in *CCU* patients, a different pattern of interactions emerged, characterised by the strengthening of linear relationships of P–T hormones, identifying a recalibration of the P–T hormone homeostatic equilibria (set points). Repeating the same analysis on groups of patients dichotomised according to low or high IL**-**6 or ferritin levels yielded similar results, suggesting that the hyper**-**inflammatory response, which is most prominent in critically ill patients, might influence this distinct pattern of P–T hormone interactions. Our data is consistent with the activation of the TSH**-**T3**-**shunt^42^ under the influence of high IL**-**6 levels and/or iron deficiency anaemia, which is thought to facilitate fT3 stability against variations in the glandular T4 output. Therefore, it appears that in this ‘hyper**-**inflammatory’**-**critical illness group of patients, inhibition of peripheral T4 to T3 conversion and resetting of pituitary TSH release point orchestrates adaptive responses so the thyroid drifts towards a new low**-**fT3 allostatic (dis)equilibrium^7^ that might indicate irreversible disruption of homeostasis associated with adverse health outcomes, as many studies identified this with poor prognosis^10,11,43^. It is noteworthy that in plots of the two ‘levers’ of P–T axis activity (fT3/fT4 vs fT4/TSH characterising pituitary influence on thyroid hormone release and conversion of fT4 to fT3), both groups of patients had indistinguishable coordinated responses. This raises the possibility of comparable adaptation despite the disruption of the homeostatic mechanisms that integrate central and peripheral control of thyroid activity, possibly under the influence of IL**-**6 or anaemia, or other systemic mediators of severe or prolonged illness.

The study analysed real**-**world biomarker data used for the routine care of admitted patients with COVID**-**19 disease. Biomarker selections were determined by requests received from clinical teams. Therefore, no research biomarkers such as levels of rT3 were available; this limited in**-**depth assessment of thyroid homeostatic mechanisms and deiodinase activity associated with NTIS^44^. Another limitation was the unavailability of extensive data around patient characteristics and biomarkers such as TPOAb that potentially might influence associations between thyroid hormones; previous population cohort studies from Rotterdam and Busselton demonstrated that age, sex, BMI, smoking, genetic determinants, and TPOAb levels influence TSH and fT4 levels as well as the relation between TSH and fT4^44,45^. We also lacked access to patient records, disease manifestations, and clinical management details. ICU medications like glucocorticoids, dopamine, and heparin can impact thyroid function^46^. Endogenous glucocorticoids, even at physiological levels, are known to affect serum TSH levels mainly by inhibiting thyrotropin**-**releasing hormone secretion in the hypothalamus or by suppressing TSH release in pituitary thyrotroph cells. Therefore, any marked elevations in endogenous cortisol secretion during COVID**-**19 could influence TSH**-**thyroid hormone associations^47^. However, as most of the findings of our outcome**-**agnostic approach were in agreement with previous biomarker studies employing an outcome**-**focused design, this suggests that our correlation**-**based data analysis approach can be used for further biomarker studies enriched by clinical outcome data. Finally, our analysis should be interpreted with caution, especially in patients with low albumin levels, as severe hypoalbuminaemia can lead to analytical errors in the measurement of free thyroid hormone (fT4/fT3) levels from one**-**step direct analogue immunoassays that are used by the majority of laboratories for the determination of free thyroid hormone^48^.

The pathogenesis of thyroid dysfunction post**-**COVID**-**19 is not completely understood. In a cohort of patients where NTIS appeared to be a frequent thyroid abnormality, our study uncovered important differences in biomarker networks and thyroid hormone adaptive responses orchestrated by inflammatory signals such as IL**-**6, especially in critically ill COVID**-**19 patients. In this example of a systemic illness often driven by an inflammatory insult, these important type 1 allostatic responses alter the P–T control loop to switch to a different operating mode with changes in set point regulation, and at the organ level, in terms of local metabolism of thyroid hormones. Whether these changes in critically ill patients are beneficial or harmful in terms of outcome probably depends on disease stage and severity, the need for long**-**term vital support, and environmental factors. Our study also highlights the importance of reviewing the approach to thyroid function test interpretation, considering the dynamic and adaptive characteristics of interrelationships between TSH and thyroid hormones, and the interlocking elements of the control system.

## Methods

### Statistical analysis

Given that the biomarkers in our dataset contain outliers and their distributions are skewed, Spearman’s correlation coefficient,* ρ*, was employed, which effectively mitigates the influence of outliers by ranking the data and focusing on the monotonic nature of the relationship rather than its linearity, thereby providing a more robust and reliable measure of association under these conditions.

Correlation heatmaps with dendrograms and scatter plots were developed to visualise the correlations between biomarkers. Dendrograms helped to identify similar patterns (clusters) of biomarker correlations. To compare the biomarker distributions between the groups of interest, i.e. the *ward* (non**-**severe disease) and *CCU* (severe disease) cohorts, boxplots and nonparametric Wilcoxon rank/Mann–Whitney U**-**test were used. The null hypothesis of no difference between the groups was tested at the 5% level of significance, and this is presented by *p***-**values. Only moderate and strong correlations (*ρ* ≥ 0.4) were considered to identify biomarkers that correlate with thyroid biomarkers, and circos diagrams were used to visualise the correlations. The false discovery rate correction was performed due to the possibility of a type 1 error.

## Supplementary Information

Below is the link to the electronic supplementary material.

Supplementary Material 1

## Data Availability

The authors will consider genuine requests for data access. For access, please contact the corresponding author at n.khovanova@warwick.ac.uk.
