# Supplementary material for: Dissecting biomarker networks linking COVID-19 inflammatory drivers, disease severity and thyroid adaptive responses
Source: Sci Rep. 2025 Nov 24;15:41542. doi: 10.1038/s41598-025-25434-1 (PMC12644497; doi:10.1038/s41598-025-25434-1)
Supplement: Supplementary file 1 — Supplementary Material 1 [file 41598_2025_25434_MOESM1_ESM.pdf]

Dissecting biomarker networks linking COVID-19 inflammatory drivers, disease severity and thyroid adaptive responses

Assem Aimaganova, Natalia Khovanova, Emma Braybrook, Evangelos Vryonis, Neil Anderson, Lawrence Young, Dimitris Grammatopoulos

SUPPLEMENTAL INFORMATION

Materials and Methods

Biomarker data descriptions and analysis

This study analysed routine biomarkers of patients from two distinct patient settings characteristic of disease severity (general *wards* and *CCU*). As the study analysed real-world data that did not follow a stringent research protocol, there was a wide variation in the number of biomarker measurements per patient that was included in the study: approximately 86% of included patients had single requests, whereas the remaining 14% had 2-8 requests per patient. Moreover, in 41% of requests, some but not all biomarkers were reported due to no request by the clinical teams or various analytical issues, e.g. haemolysis, insufficient sample, or reagents availability. For example, TSH results were available in 575 specimens, fT4 in 396, and fT3 in 322, reflecting differences in routine clinical practice. There were 237 complete records, which were included in the analyses concerning the *ward* and *CCU* groups. The fT3 to fT4 ratio (fT3/fT4) was calculated to provide information about thyroid hormone metabolism and the degree of conversion of T4 to T3, a process influenced by nutrient availability and hormonal factors[1]. The final dataset included 31 different biomarkers: 27 COVID biomarkers, 3 thyroid function tests (TFT), and fT3/fT4. Unlike other studies, we did not restrict data analysis by comparing associations with comorbidities such as diabetes, hypertension, chronic kidney disease, cardiovascular disease, endocrine disease, current diagnosis of cancer at the time of admission, obstructive pulmonary disease, including asthma, and chronic obstructive pulmonary disease. Moreover, exclusion criteria included patients with a documented previous history of thyroid disease and/or those who were taking thyroid hormones or antithyroid medications.

The biomarkers collected were measured at the Diagnostic Pathology laboratory, UHCW NHS Trust, a UK accreditation service (UKAS)–accredited laboratory, during patients' stay at the hospital. All in-patient specimens were collected between April 2020 and October 2022 and

tested for determination of biomarker parameters on a Roche Cobas e602 immunochemistry module using Elecsys immunoassays (for TFTs, troponin-T (TNT), ferritin (FER), IL-6 and procalcitonin (PCT)) or the 702 chemistry module employing photometric (for lactate dehydrogenase (LDH), albumin (ALB), iron) or immunoturbidimetric assays (for transferrin (TF)). Haematology parameters (haemoglobin (HB), platelets, neutrophils, lymphocytes) were quantified in a Sysmex XE-2100 platform; HB was measured using the sodium lauryl sulphate spectrophotometric detection method, whereas platelets, neutrophils and lymphocytes were measured by flow cytometry.

REFERENCES

1. Holtorf, K. Peripheral Thyroid Hormone Conversion and Its Impact on TSH and Metabolic Activity Journal of Restorative Medicine 2014; 3: page 31 Peripheral Thyroid Hormone Conversion and Its Impact on TSH and Metabolic Activity. *J Restor Med* 3, 30 (2014).

Table S1. Biomarker characteristics.

N	Biomarker name	Sample size	Min value	Max value	Mean	Median
1	TSH, mIU/L	575	0.02	86.26	2.21	1.35
2	Free T4 (fT4), pmol/L	396	5	53.4	16.93	16.50
3	Free T3 (fT3), pmol/L	322	1.5	9.2	3.29	3.20
4	*fT3 to fT4 ratio (fT3/fT4)*	318	0.07	0.44	0.2	0.19
5	Procalcitonin (PCT), ug/L	575	0.06	100	1.53	0.16
6	Interleukin-6 (IL-6), ng/L	575	2	5000	258.83	44.00
7	C-reactive protein (CRP), mg/L	575	3	424	75.15	47.00
8	Albumin (ALB), g/L	575	11	52	32.85	33.00
9	Ferritin (FER), µg/L	574	17	19604	1081.93	657.00
10	Transferrin (TF), g/L	574	0.5	3.9	1.80	1.80
11	Iron, µmol/L	570	1	43	9.28	7.00
12	Troponin T (TNT), ng/L	563	5	2239	55.92	23.00
13	Lactate dehydrogenase (LDH), U/L	504	106	10736	533.05	438.00
14	Sodium, mmol/L	501	115	161	138.93	139.00
15	Urea, mmol/L	501	1.10	42.90	9.77	7.60
16	Creatinine (CRE), µmol/L	501	21	1253	102.31	75.00
17	Potassium, mmol/L	492	2	7.50	4.39	4.40
18	Mean corpuscular volume (MCV), fL	484	56.70	120.40	91.12	91.25
19	Mean corpuscular haemoglobin (MCH), pg	484	17.50	38.90	29.21	29.30
20	Platelets, ×10^9/L	484	17	876	282.07	269.50
21	Haemoglobin (HB), g/dL	484	66	180	118.99	120.00
22	Haematocrit (HCT)	484	0.21	0.55	0.37	0.38
23	Red blood cells (RBC), ×10^12/L	484	2	6.59	4.09	4.15
24	RBC distribution width (RDW), %	484	11.40	26.20	14.94	14.50
25	White cell count (WCC), ×10^9/L	483	0.93	148.20	12.06	9.69
26	Monocyte, ×10^9/L	483	0.04	5.25	0.60	0.52
27	Neutrophil, %	483	0.6	33	8.63	7.69
28	Lymphocyte, %	483	0.12	120.49	2.47	0.98
29	*Neutrophil to lymphocyte ratio (NLR)*	483	0.08	137.31	11.62	7.45
30	Basophils, ×10^9/L	439	0.01	4.28	0.07	0.02
31	Eosinophils, ×10^9/L	370	0.01	2.04	0.15	0.07

Table S2. Demographics description of 237 samples.

	Age, mean (range)	Female	Male	Anaemia
***CCU* (59 %)**	57 (21-79)	44 (31 %)	96 (69 %)	87 (62 %)
***Ward* (41 %)**	63 (20-95)	40 (41 %)	57 (59 %)	45 (46 %)

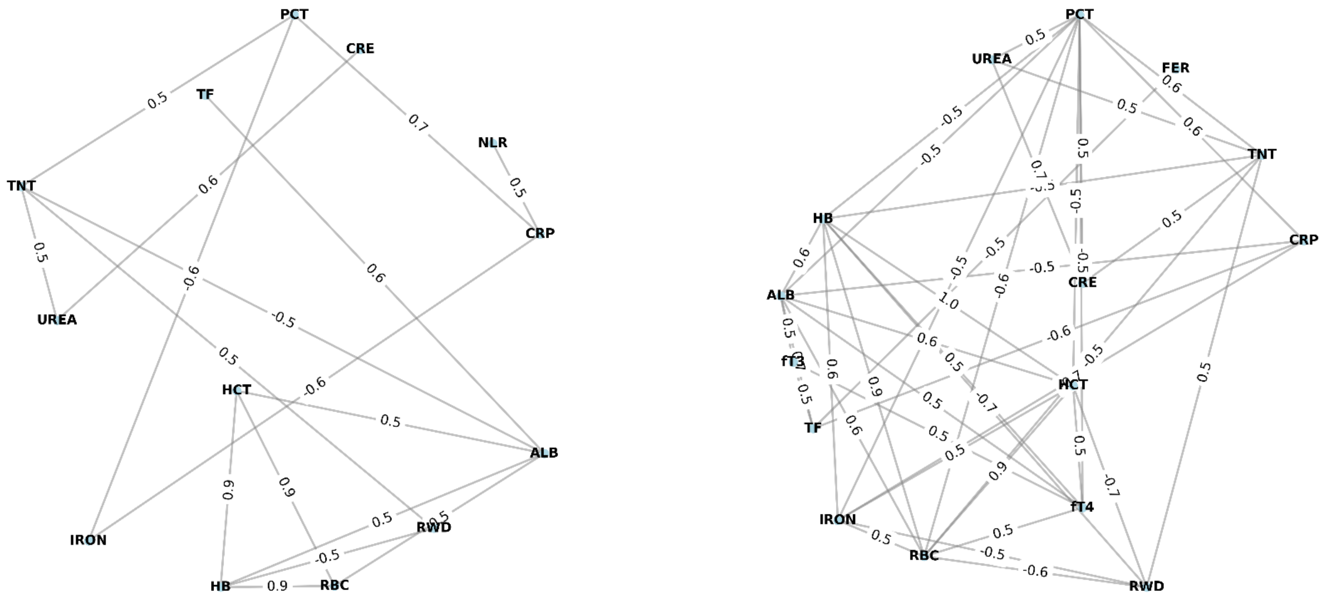

Figure S1. Network diagrams showing the biomarkers that have moderate and strong correlations in the *ward* (left) and *CCU* (right) patients. Significant correlations across a cluster of 13 biomarkers were detected in the *ward* group. In contrast, 39 correlations across 15 biomarkers (the same cluster enriched by 2 thyroid hormones and ferritin replacing NLR) were present in the *CCU* group.

a

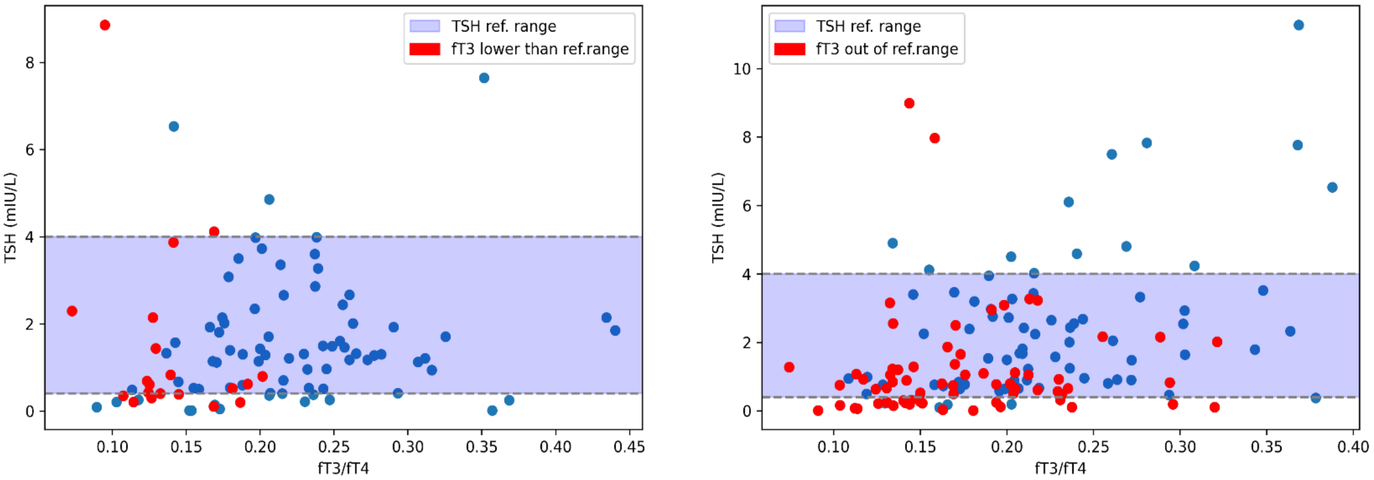

b

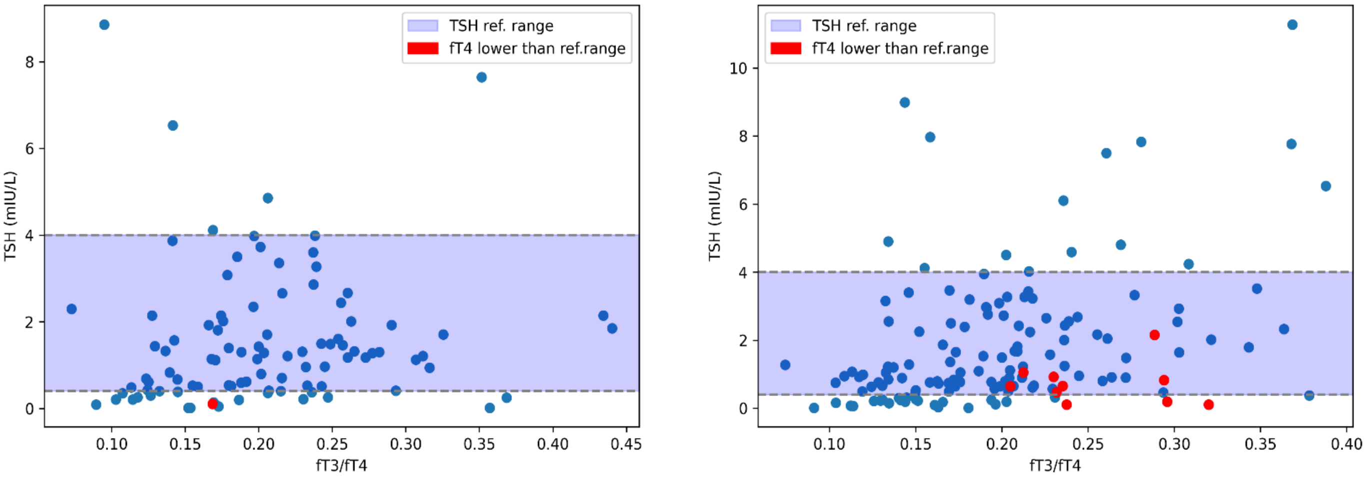

Figure S2. (a) Scatter plot of TSH vs fT3:fT4 ratio in the *ward* (left) and the *CCU* (right); red points indicate low fT3 values. (b) Scatter plot of TSH vs fT3:fT4 ratio in the *ward* (left) and the *CCU* (right); red points indicate low fT4 values.
